# Roles of health promotion researchers in the planning stages of a global urban health promotion initiative: understandings identified from an interview-based case study

**DOI:** 10.3389/fpubh.2025.1574732

**Published:** 2025-05-21

**Authors:** Sophie Meyer, Nathalia González-Jaramillo, Annika Frahsa

**Affiliations:** ^1^Institute of Social and Preventive Medicine, Faculty of Medicine, University of Bern, Bern, Switzerland; ^2^Graduate School for Health Sciences, University of Bern, Bern, Switzerland; ^3^Multidisciplinary Center for Infectious Diseases, University of Bern, Bern, Switzerland

**Keywords:** researcher role perception, science-policy-interaction, governance for health, health promotion research, epistemology-formation and academic production, qualitative content analysis, social science, researcher reflexivity

## Abstract

**Introduction:**

Health promotion research is marked by recognizing diverse forms of knowledge, the embeddedness of research practices in context, the relationship between researchers and stakeholders, and the articulation of knowledge production and sharing. Amid this epistemology, researchers’ understanding of their roles in specific projects and programs led by different stakeholders is essential. We used a global initiative to promote governance for health and wellbeing in five cities of different low-and middle-income countries as a case study to analyze senior-level researchers’ understanding of their role within the initiative.

**Methods:**

We conducted a qualitative content analysis, supported by computer-assisted qualitative data analysis software, of verbatim interview transcripts from semi-structured qualitative interviews with the full sample of senior-level health promotion researchers (*n* = 5) who supported implementation of the initiative.

**Results:**

We identified three diverging types of local researchers’ roles understandings: (1) active deep involvement in collaborative arrangements, (2) balancing between active involvement and passively supporting, and (3) passively supporting the initiative. Researchers transcended sectoral boundaries to varying degrees and acted at the nexus between academic, practice, and policy communities.

**Discussion:**

Our proposed typology delineating the roles of senior-level health promotion researchers has the potential to stimulate reflexivity regarding role comprehension and underlying assumptions among all stakeholders before and during the implementation of ongoing and future urban health initiatives.

## Introduction

Tackling complex health issues—especially in urban, low-resource settings—requires collaborative, context-responsive approaches that span disciplines, sectors, and systems. Various research traditions like public health research (1–3) and environmental or sustainability science (4) have emphasized the need to work transdisciplinarily across knowledge domains and organizational boundaries. Similarly, research at the science–policy interface (5) and in multisectoral health partnerships (6) highlights the increasingly strategic role researchers play in both shaping and supporting interventions.

Health promotion research covers a diverse range of research methods shaped by different epistemological perspectives. Despite this diversity, common knowledge-related themes emerge across various research practices. These recurring themes help outline an epistemological framework for the field, not by enforcing uniformity but by highlighting key issues influencing how research is conducted and understood. Potvin and Jourdan suggest four shared epistemological foundations that may guide researchers in their diverse research practices (7): (1) Recognition of diverse forms of knowledge, (2) embeddedness of research practices in context, (3) relationship between researchers and stakeholders, and (4) articulation of knowledge production and sharing.

Amid these markers, researchers’ understanding of their specific role is essential in the functions they assume in interventions and innovations, in relationships with other stakeholders, and in the actions they take to render knowledge usable by practitioners. Following Potvin and Jourdan, researchers can lead the intervention’s design and implementation, or they can conduct research to support and derive knowledge from actions led by non-research organizations (7). In the context of science and policy interaction in health, Lavis et al. (8) similarly addressed two traditional knowledge exchange modes: *knowledge pull*—where knowledge users “pulled” evidence from research to support a specific (policy) aim—and *knowledge push*—where scientific evidence is actively “pushed” out to decision-makers or knowledge users. These dichotomous, somewhat linear concepts are complemented by approaches focusing on shared knowledge creation, e.g., described by Rütten et al. (9) as the *interactive approach*—in which initiatives are co-created through a trustful, collaborative relationship between different relevant actor groups. When advising policymakers, researchers can provide descriptive problem advice to provide decision-makers with information about the existence and dimension of a problem rather than discussing solutions (10). It has been promoted in the literature that researchers may assume multiple roles in intervention and policy contexts—for instance, Ballard et al. (11) distinguish roles such as substantive expert, change agent, and information processor in applied social research, while Pielke (12) outlines archetypes like the honest broker and issue advocate to describe scientists’ engagement at the science-policy interface.

Considering the diverse “collaborative arrangements with local actors and decision-makers,” (7) health promotion researchers play an essential role in health initiatives and interventions: they integrate various types of knowledge into their research. Furthermore, researchers and their insights are inevitably embedded in the respective social contexts.

Indeed, many published articles push the presented evidence with policy recommendations to practitioners and policymakers. This is when researchers lead innovation and push for an initiative (13). Others focus on researcher roles’ supporting innovation with knowledge derived from research to support innovation and initiatives by non-research organizations (14, 15). Efforts to map practices of health promotion research (16) have not yet comprehensively covered the spectrum of academic researchers’ roles, particularly their emic understanding of their role in complex interventions. There remains a need for empirical studies that explore and structure the diverse contributions of health promotion researchers in intervention and implementation processes.

To address this empirical gap and investigate the role understanding of senior-level health promotion researchers in a large-scale urban health initiative, we present a case study of the role of senior-level health promotion researchers in an urban health initiative to promote health and wellbeing in five cities in different low-and middle-income countries (LMIC). We ask: how do senior-level health promotion researchers understand their roles, challenges, and differing expectations within a large-scale urban health initiative across five cities in low-and middle-income countries? We illustrate the four epistemological markers of health promotion research by Potvin and Jourdan (7) with empirical data in a non-research-driven initiative.

## Methods

### Study setting

Our study was conducted within a global urban health initiative (2021–2028). We accompanied the initiation and onboarding phase of the initiative from May 2021 to October 2023. In this study, we present findings about health promotion researchers understanding of their role during the setup of the initiative, which took place prior to the planning and implementation of local projects. The initiative aimed to improve the health and wellbeing of populations in urban settings through community engagement and multisectoral urban governance. It furthermore planned to establish an institutional and policy framework for effective urban governance of health and well-being at local, national, regional, and global levels, with a strengthened capacity of local and national governments and evidence supporting urban health governance. Additionally, the mayors of the participating cities committed themselves to enhancing participatory urban governance by strengthening multisectoral collaboration, fostering community engagement, and promoting social innovation to address key priorities in urban health. The initiative was led by a global health organization’s main office and operated at regional and country levels with five research partners on the ground.

Implementation was planned in five cities of different LMIC, as defined by the World Bank (17): Bangladesh, Cameroon, Colombia, Mexico, and Tunisia. While the initiative was global in nature, we explored the initiation of project implementation and the planning phase of the operational research at local levels in these five cities.

### Approach

Our study followed an explanatory case study design (18). We worked within a qualitative paradigm and from a constructivist epistemological standpoint.

We conducted an analysis of qualitative data collected in the initiative. We interviewed the full sample of the initiative’s senior-level researchers appointed by the global health organization (*n* = 5). The qualitative paradigm emphasizes rich, in-depth descriptions gathered through interviews or observations, among others. It is often interpretive and flexible, allowing researchers to explore complex social realities rather than seeking universal, objective truths. Linked to this, a constructivist epistemological standpoint is interested in how knowledge is formed, arguing that knowledge is socially constructed rather than objectively discovered. Furthermore, this construction has a reciprocal relationship between the researcher and the participant (19).

### Researcher positionality

The authors include three female researchers of Colombian, German, and Swiss Nationality. One senior researcher is a social scientist experienced in community-based participatory health promotion research and policy analysis, one researcher was, at that time, a PhD candidate (MD with specialization in epidemiology and background in research and development, with several years of research experience in Colombia) and, one junior researcher a PhD candidate specialized in science-policy interaction and a humanities background. Two authors (AF and SM) had extensive experience in conducting qualitative research. The authors are multilingual researchers fluent in English, German, French, and Spanish. We gained insights from a second-order observation perspective (20, 21) in which we sought to “maintain a balance between being an insider” and “being an outsider” (22): we were mandated by the same contracting authority as our interviewees, and we conducted research about the initiative. Whereas authors AF led and NG managed the global research component of the initiative, author SM joined meetings with different constellations of participants (initiative leads, researchers, country and regional bureaus). When AF moderated or presented, either NG or SM or both NG and SM participated as observers. Throughout the study, we reflected upon our position—especially our position toward our interviewees who were simultaneously collaborating partners—through minutes and field notes taken during meetings and introspective discussions after meetings.

### Interviews

In 2021, we conducted semi-structured qualitative interviews (23) with all designated researchers (*n* = 5) from the participating cities in the initiative. We assigned pseudonyms to participants—Arthur, Maria, Nadia, Paul, and Evan—to protect their anonymity. Given the geographical spread of the initiative and the prevailing COVID-19 pandemic at that time, we conducted all interviews online through Microsoft (MS) Teams^®^. We interviewed participants either in home office settings or workplaces. Interviewees had different specializations, such as urban planning, physical activity, or epidemiology. We sent participants a short study description and interview topics prior to the interviews. SM piloted and revised the initial interview guide. Interviewees were asked about their role within the initiative, insights, experiences with, and ideas about implementation plans of community engagement and multisectoral action in their respective cities. We also asked about their experiences in science-policy exchange. All questions were open-ended, with several sub-questions to deepen the inquiry and be flexible enough to follow the participant’s narrations. The interview guide is accessible as Supplementary material S1. The longest interview lasted 53 min, the shortest 22 min (mean = 43 min).

We followed a transcription guideline and transcribed interviews verbatim. We pseudonymized transcripts and stored all original data on password-protected servers at the University of Bern.

### Data analysis

We followed qualitative content analysis (24) supported by computer-assisted qualitative data analysis software (MAXQDA2020). In analyzing the data, we blended an initial interpretivist coding (researchers’ meaning-making of their role) with a constructivist sensemaking in results (construction of types by authors based on interviews).

For category development, we employed both deductive and inductive approaches (24). During the coding process, we held multiple meetings to discuss codes and preliminary concepts. We applied classic barriers and facilitators analysis and science-policy interface theories [following (25, 26)] as sensitizing concepts. Inspired by Weber’s heuristic device of the ideal type (27–29), we constructed three different types of understanding of researchers’ roles in the initiative. We developed those types based on concepts and codes applied in the content analysis and meetings of critical friends (30). In the categorization, we aligned the ideal types according to the third marker of the epistemological framework in health promotion research: “relationship between researchers and other stakeholders” (7).

## Results

We first present the demands researchers faced by the contracting authority; second, relevant contextual factors of overall collaboration within the initiative; third, framed by these findings, we distinguish three types of understanding of researchers’ roles in the initiative: (1) active *deep involvement in collaborative arrangements*, (2) *balancing* between active involvement and reactive supporting, and (3) passively *supporting* the initiative.

### Defining the researchers’ role: terms of reference by the contracting authority

Terms of Reference (TOR) developed by the main office of the contracting authority served as the base for national or regional offices to appoint research partners. TOR included information for adapting and implementing a research and evaluation protocol in respective cities. Research partners were commissioned to

1. conduct desk reviews on context-specific evidence,
2. customize—and subsequently implement—available research, monitoring, and evaluation tools for local contexts and
3. conduct original research through data collection and analysis, including reporting results, lessons learned, and case examples.

In addition, they were to

4. facilitate exchanges with global academic research institutions,
5. facilitate and participate in a training of trainers program, including representatives from municipalities, academic partners, and civil society groups, and
6. promote and engage in collaboration with and capacity building among stakeholders.

Researchers were appointed to oversee and facilitate meetings within a leadership program to engage stakeholders from all participating cities for capacity-building purposes. Their primary responsibility entailed establishing, nurturing, and maintaining visible networks. In preparation for presenting their initiatives during these meetings, they adapted global instruments to their respective local contexts and deployed these research tools on the field. However, once meetings grew more comprehensive in scope—and city mayors attended—leadership shifted back to the contracting authority. With its broad scope of tasks, the TOR allowed for individual prioritization of specific tasks, which is also reflected in different types of understanding of researchers’ roles. Before we describe each of these roles in detail, it is important to provide the contextual background against which they should be understood.

### Contextual factors

While organizational structures and hierarchies may streamline administrative procedures, in this study, top-down management and delegated responsibilities posed challenges for staff and external partners. Bureaucratic contract finalizations clashed with hastily arranged online meetings, limited preparation time, and large participant numbers. External factors, including COVID-19 delays and political and environmental crises in participating countries, exacerbated the situation. Despite written agreements about collaboration from the initiative’s lead, researchers reported they were unclear about work packages and concrete tasks and work packages or the overall aims of the initiative. For example, Arthur stated,

I think, at this point, everybody is a little bit confused about what the project is about, what their role is supposed to be, and how we are supposed to do things right.

It also led to a perception of being only loosely affiliated with the initiative, as pointed out by Maria:

I have been in this interview and sometimes on a call that [the head of the regional bureau] has, but nothing official.”

In some cases, unconfirmed and very short-term contracts provoked perceptions about the non-official affiliation, which resulted in either perception of overly ambitious deliverables or low project commitment. It is against this background of challenging work situations and somewhat vaguely described tasks that the following classification is to be understood. Researchers emphasized the planning and presentation of the desk review or small on-site programs; others balanced between evidence creation and deep involvement in collaborative arrangements or pushed for action as good as the circumstances permitted them. Based on those findings, we distinguish three types of understanding of researchers’ roles: (1) deep involvement in collaborative arrangements, (2) balancing, and (3) supporting.

### Deep involvement in collaborative arrangements

The *deeply involved* type actively engaged stakeholders, analyzed the field, and rendered knowledge usable by practitioners, embodying a hands-on interactive approach known as co-production-rather than knowledge push. This type focused on identifying decision-makers, understanding organizational and political structures, inclusivity of voices, collaborative partnerships, and assessing the city’s health status quo.

Paul and Evan represented the leading type. Evan—part of a larger team of researchers involved in this city—took the lead and (re)presented the whole team with various activities, such as stakeholder engagement actions, awareness-raising activities, and memoranda of understanding with community leaders. Evan positioned himself in a nexus role through high visibility during several meetings, presentations, and discussions, bringing in his expertise as a trained physician and health policy expert. Paul stated,

We are eager to get started, […] we’ve been expecting the contract to be signed so that we can get into the field and having access, actually, to some of the decision-makers to have their own perspective on the project.

While working on data collection and analysis, the deeply involved type emphasized engaging in exchanges, capacity building, and collaborating with stakeholders, such as academic partners, municipalities, and civil society groups.

Contrary to other researchers in the initiative, Evan and Paul were very active despite short-term contracts. Even amid uncertainty and confusion, their belief in the initiative and its outcomes was tangible. They demonstrated considerable effort toward commissioned work, such as broader, far-reaching tasks. In acting as multipliers, researchers were assigned to “train local champions” or train the trainers. And thus, they bore the responsibility for functioning implementation networks.

Designated researchers were both present on the ground, and they were city locals in most cases. Interviewees repeatedly informed us about the specifics of city contexts. They emphasized peculiarities to consider when planning and implementing initiatives in their city related to governmental structures and political processes, such as decentralization, or when cultural characteristics shaped interpersonal interactions. Using their awareness of politicians’ influence as well as shared past worksites or employers with decisionmakers, they actively linked actors with each other whom they deemed potentially relevant to the initiative. Evan explained,

[…] everybody knows the mayor, and the mayor is a former minister. […] This is the opportunity we need to use if we really initiate a healthy city initiative involving all the stakeholders with a new mindset that this will have to do in a collaborative platform.

### Balancing

The *balancing* type resided between supporting and leading, characterized by a clear interest in science-policy exchange but not clear regarding knowledge push to targeted users or interactions with decision-makers. Whereas the *deeply involved* type was active in several aspects of the initiative and the *supporting* type was rather passively assisting where the intervening institution asked for it, the *balancing* type balanced between these two engagement poles.

Nadia represented the balancing type – currently a public health academic with a formerly high position at a public health department who understands both roles: knowledge user and knowledge producer. Despite such an ideal starting point for deep involvement in collaboration arrangements, she understood her role as a researcher with the task of developing and implementing research projects within the initiative. Doing so, Nadia walked the line between support and being deeply involved, producing basic evidence through research projects, as well as displaying an urge for actual implementation through collaboration with governments and decision-makers. At the same time, she showed disappointment about the non-consideration of scientific evidence and researchers in the current political administration. Nadia explained,

We publish a lot of papers about many aspects of NCDs [non-communicable diseases]. But the ministry of health […] don’t refer to these papers. They just discuss with the directors, the local and the directors of the district. They don’t refer to academia.

### Supporting

The understanding of researchers’ role as *supporting* type passively focused on assisting the intervening institution in a knowledge-pull-environment. Such an understanding maintained and supported a perceived division between knowledge producer—academia—and knowledge user—public administration and policymakers. Their main competencies are perceived in data collection and evidence creation, thus moving away from the overall epistemological framework of health promotion research, particularly from collaboratively changing health-promoting properties of whole systems and knowledge-sharing with practitioners. The supporting type fulfilled its tasks in the initiative through the provision of scientific problem advice.

Arthur and Maria represented the supporting type in their understandings of their roles. Onboarded by the health organization’s regional bureaus, they worked to support the initiative with potential research projects and evidence. They viewed themselves as participants in knowledge production activities and contacted knowledge users only if needed for knowledge production. They reflected, nevertheless, about how to best generate awareness within governments for the overall topics of the initiative and aimed to increase policymakers’ understanding of the width of urban health, as Maria indicated:

I think it will be important to help our policy-makers to understand the importance of what they do in health. I think they do urban health. I mean what they do is all urban health, but I do not think they realize that. So this project, it would be nice to help them in their discourse in what they talk to implement health.

Still, we noted a “clear divisional line” between academia and policy when it came to implementation. For instance, Arthur explained

I think it is going to be very important for us to understand exactly what they want to do and how do they want to do it. It is very unrealistic to think that we [academia] are going to teach them [policy-makers] how to do things.

Similarly, Maria pointed out the different logics of policy-making and research, with the former potentially profiting from considering urban health research insights and an urban health lens in concrete actions.

Maria and Arthur shared a perception of neither taking part explicitly in policy-making nor implementing the project. They understood their roles as—first and foremost—conducting a baseline study within the initiative. Their understanding mirrored the axiom of science as evidence producing, processing, or evaluating—i.e., the knowledge pull mechanism. Furthermore, such understanding was sometimes accompanied by hierarchical notions of the government-academia relationship, whereas the latter appeared minor when compared with the former. Contrary to the deeply involved type, overdue contract signing and scarce financial resources for conducting commissioned research hampered engagement for the supporting types. For instance, Arthur asserted

I think from our side it is very important to have a very clear idea of what it is that you are expecting from us and also to make sure that what is being expected from us is proportional to the level of support that we get to do the project. Because it is very difficult for us to sustain an effort with no funding for instance.

### Science-policy interaction in perspective

Across all three types, science-policy exchange was perceived as a highly important topic. Nadia viewed academia and policy as “two separate worlds” that neither collaborate nor communicate. Interestingly, Maria, Arthur, and Nadia reported a solution to science-policy exchange—personnel exchange between academia and ministries—which made exchange easier and cooperation more pleasant. Paul described the interaction as shaped by “a lot of informal connection between university and policy-makers.”

Even if there existed links between science and policy, they appeared to be rather weak or unsustainable. Several researchers described a need for institutionalized exchange structures. For example, Evan aimed for a collaborative platform at the city-level with clearly defined mandates and active participation from stakeholders. Arthur depicted the same situation yet preferred a structured process for exchange between researchers and policy-makers:

Despite that there is not a permanent space where we can exchange ideas and participate in the development of health initiatives or programs. That does not mean that they do not have their own advisors, and they probably do. I am just not one of them. But I think in general it could be very good if we could create like a much more sustainable space, where perhaps the government poses a question or poses a problem, and then the academia tries to chip in to help solve some of that problem in a much more permanent way. Like creating a sort of a specific space to exchange ideas between academia and the government.

To achieve urban health promotion, the initiative required close collaboration between researchers and stakeholders on the ground as well as scientific evidence based on the current status quo to guide decision-making on changes and outcomes to achieve with the initiative. Within this context, the supporting type emphasized evidence production tasks assigned to them, such as conducting desk reviews of existing local or national evidence on urban health and compiling basic scientific research in their city contexts for improving understanding of local barriers and facilitators. The balancing type emphasized the need for implementation, though they took no action to push for it, and the deeply involved type focused on engaging stakeholders and rendering knowledge usable by practitioners.

In sum, research partners in the initiative to promote health and wellbeing in five different cities varied considerably regarding the markers of the epistemological framework in health promotion research, particularly regarding stakeholder engagement, which varied from active through cautious to limited stakeholder engagement. This variation appeared to be amplified by different pre-set structures for knowledge exchange in the different participating cities. In some cities, exchange was moderated and supervised by the global health organization’s regional office (related to Maria’s and Arthur’s contexts). Researchers had already established points of contact in other cities and moved forward from there (Evan, Nadia).

## Discussion

Our study investigated the expectations, demands, and role understandings of senior health promotion researchers within a global urban health initiative in five cities of low-and middle-income countries. With this, we address the lack of empirical research on the broader spectrum of health promotion researchers’ roles in giving attention to how researchers understand their roles when planning complex interventions. Our study helps to deepen understanding of the diverse contributions and practices of health promotion researchers in innovation processes. We found some health promotion researchers to be firmly based on the epistemological markers of health promotion research. Deeply involved researchers recognize and value diverse forms of knowledge when working collaboratively with communities and policy makers. They are skilled with contextual—sometimes tacit—knowledge and acknowledge the embeddedness in their research contexts. They furthermore added the “necessary human element of interaction” (31) required for effective implementation practice. We also found various factors hindering the smooth collaboration of non-research stakeholders and researchers despite a given understanding by researchers as being deeply involved. Challenges included conflicts between predefined roles and actual implementation, also highlighted by other studies that focused on the concept of knowledge brokers (32). Health promotion researchers, particularly in complex systems like global urban health initiatives, may face difficulties in defining tasks due to diverse stakeholder needs and perspectives. The initiative itself demonstrated complexity linked to political, social, and financial factors, yielding a non-linear and unpredictable trajectory, thereby posing multifold governance challenges. Conscious project creation and design could mitigate these issues (33). This complexity aligns with insights from public health, environmental sciences, and sustainability research, where transdisciplinary collaboration, stakeholder engagement, and context-responsiveness are emphasized as key to successful intervention (1, 2, 4).

We also identified researchers in the initiative in mostly knowledge production and supporting roles. This variability in engagement aligns with Potvin and Jourdan’s remark that certain research projects or programs align more naturally with some markers than with others and that also individual “researchers may be more or less explicit in their respective position on several or all of these markers” (7). We presented three types of understandings of health promotion researchers’ roles within the initiative. These roles relate to different concepts: some researchers assumed a rather neutral role in evidence production and knowledge translation, being a balancing and a more reactively supporting the initiative. Others understood their role as deep involvement in a co-creation process ranging from deep involvement in co-creation and assumed capacity to influence the overall setup of the initiative and overcoming disciplinary work. Furthermore, other typologies described in the literature (11, 12) suggest that researchers’ involvement in interventions and policymaking is linked to context, policy goals, and disciplinary norms.

This diversity of role understanding within the same urban health initiative underscores the complexity of role understandings in such initiatives led by non-research organizations. We make a case for the necessity for health promotion researchers to at least reflect the four markers of the epistemological framework for health promotion research, especially in projects or programs led by non-research organizations that aim to foster urban governance for health and wellbeing.

Researchers’ work within the initiative took place on the margins of scientific research. This calls for researchers’ reflexivity on roles, values, and accountability. Researchers’ reflexivity focuses on the relationship between researcher and subject, the role of subjectivity in the field, and its reflection in scientific work (34). Reflexivity is particularly significant in transdisciplinary and transformative research, as such research challenges the existing values, assumptions, and power structures shaping scientific organizations and their epistemological models (35). We argue that reflexivity is also decisive when working with non-academia partners in projects and programs aiming at real-world change. In these endeavors, researchers may face tensions, for instance, in modes of knowledge production when moving away from purely descriptive approaches toward actively supporting processes of change. To address such tensions, researchers’ reflexivity has been suggested as “a coping strategy for dealing with complexity and ambiguity” [(35) p. 17].

We suggest that contracting authorities further support the deeply involved research partners in global urban health initiatives through (1) clearly defined mandates, (2) appropriate financial resources, and (3) administrative support. This assessment agrees with key insights on successful cross-sector collaborations throughout projects, as suggested among others by van Vooren et al. (36) (1) building trust among and creating faith in project partners; (2) engaging crucial stakeholders during the entire project process; and (3) defining roles, tasks, and other prerequisites at the start of the project. Harmonizing tasks, competencies, and responsibilities may also lead to higher engagement with commissioned work. Assuming such support is given, deeply involved research partners could conduct *a priori* analysis of local implementation contexts and stakeholder assessment and invest in trust-building activities with other stakeholders. This would support large-scale interventions that aim to promote governance for health and wellbeing, in which multisectoral efforts are needed to overcome existing silos (37).

Such approaches show similarities to concepts described in the literature as knowledge brokers which might help to explicitly bridge the gap between underuse or inconsistent use of “high-quality evidence” (38) and policymaking for change. In addition to administrative enhancements, interviewees in our study proposed establishing permanent exchange platforms or forums to engage decision-makers and civil society. Collecting stakeholders’ ideas upfront could guide the development of tailored tools for future endeavors. Additionally, knowledge brokering is to be understood as a function that outlives the lifespan of specific projects. They need to be embedded and promoted through systems, for instance, policy advisory systems (5, 39).

### Strengths and limitations

We suggest interpreting our findings considering the strengths and limitations of our study. Our methodological approach allowed us to reflect upon roles within an urban health initiative; our role as participatory observants provided the opportunity for in-depth insights while simultaneously generating meta-knowledge about processes in the initiative. This allowed us to create a feedback loop for the initiative lead. Furthermore, the identification of three distinct researcher role understandings among five participants underscores the value of qualitative methods in revealing nuanced motivations and understandings.

However, our study also shows some limitations. While the typology presented in this study is based on a relatively small sample of five interviews, the typology is not intended to be exhaustive or universally transferable to all health promotion researchers. Rather it provides a foundation that could be built upon, modified, and tested in future research. Other methodological approaches, for instance, involving a larger and more diverse group of European health promotion researchers could explore the applicability of these roles, identify additional categories, or refine the typology based on broader perspectives. Collaboration heavily relies on personal connections and the influence of gatekeepers, who play a crucial role in the success of projects aiming for real-world change. This further emphasizes the importance of considering contextual factors in similar initiatives.

## Conclusion

Researcher partners in urban health initiatives work at the nexus of science, policy, and civil society, between deep involvement, balancing, and supporting functions. Our proposed typology of researcher roles within a global urban health initiative provides a framework for reflexivity about role comprehension and underlying assumptions held by both contracting authorities and research partners themselves, both in planning and implementation of future health initiatives. For the advancement of policy-science-interaction in such initiatives, further research and critical reflection is needed on defining, implementing, and evaluating partner roles.

## Data Availability

The datasets presented in this article are not readily available because all data is stored on the servers of the University of Bern. Data will be made available on reasonable request. Requests to access the datasets should be directed to annika.frahsa@unibe.ch.
