# Supplementary material for: Roles of health promotion researchers in the planning stages of a global urban health promotion initiative: understandings identified from an interview-based case study
Source: Front Public Health. 2025 May 21;13:1574732. doi: 10.3389/fpubh.2025.1574732 (PMC12133794; doi:10.3389/fpubh.2025.1574732)
Supplement: Supplementary file 1 [file Data_Sheet_1.PDF]

# Interview guide

## Welcome and introduction

My Name is …. , I am **position in project* *

On behalf of the team, I would like to thank you for your time and willingness to participate in this project. I look forward to learning about your experiences and your personal views on the matters of this interview.

I will take notes during our conversation, please do not let this distract you.

As mentioned in the consent form/in our earlier contact: I will record our conversation so the team can later transcribe it. And subsequently better summarize what we have learned from your experience.

**Do you have any questions about the interview before we start?**

If not, I will **now start the recording** and we will start the interview.

## Part 0 Introduction

In the beginning, I would like to get to know a bit more about your professional background and function. What is your concrete role when it comes to the initiative in CITY NAME?

Please tell me a bit more about how you came on board?
How did the **selection process** look like?

How would you **describe the current state of your involvement** in this initiative?

## Part I Personal Experience and insights

In this part of our interview, we would like to learn about your personal experience regarding urban governance for health, wellbeing, and equity through **community engagement** and **multisectoral action** in city NAME.

1. When you think about "urban governance for health, wellbeing and equity", what does that mean to you in CITY NAME?

**Inquiries about community engagement**

You mentioned XY…

You haven't mentioned community engagement yet…

Could you tell me a bit more about…

In your opinion…

a. …What are the specific **strengths** in CITY NAME in terms of urban governance and **community engagement**?
b. … Are there also **challenges** in CITY NAME in terms of urban governance and **community engagement**?

**Inquiries about multi sectoral action**

You mentioned XY…

You haven't mentioned multi sectoral action yet…

Could you tell me a bit more about…

In your opinion, are there…

a. … specific **strengths** in CITY NAME in terms of urban governance and **multisectoral action**?
b. … possible **barriers** in CITY NAME in terms of urban governance and **multisectoral action**?

2. Please think about the special situation of the COVID-19 pandemic. What multi sectorial actions and/or civic engagement activities come to your mind that help(ed) to tackle COVID-19 in CITY NAME?

**Inquiries**

Could you give me an **example** for this?

Could you **elaborate** a bit further?

## Part II Goals in Urban Health and Community Engagement and Multi-Sectoral Action

Now that we looked at what has been going on, we would like to understand a bit more what CITY NAME has planned for the future in terms of including multi sectoral action and community engagement strategies in its urban governance.

3. Could you tell me from your position as a representative of the government / as someone assigned to implement ideas/ as a Scientist, how do you see multi sectoral action and community engagement being incorporated in the future into urban governance for health, wellbeing and equity in CITY NAME?

**Inquiries about community engagement**

You mentioned XY…

You haven't mentioned community engagement yet…

Could you tell me a bit more about…

In your opinion…

a. What are the specific **opportunities** in CITY NAME in terms of urban governance and community engagement?
b. … What could be specific **challenges** in CITY NAME in terms of urban governance and community engagement?

**Inquiries about multi sectoral action**

You mentioned XY…

You haven't mentioned multi sectoral action yet…

Could you tell me a bit more about…

In your opinion, are there…

a. … What are specific **opportunities** in CITY NAME in terms of urban governance and multisectoral action?
b. … are there also possible **challenges** in CITY NAME in terms of urban governance and multisectoral action?

## Part III Science and Policy exchange

In this last part of our conversation, we would like to focus on one specific strand of multi sectoral action, namely **science and policy exchange** in CITY NAME.

4. How would you describe the **relationship between academia and urban governance** in CITY NAME?

**Inquiries**

Can you tell me about your previous experience when comes **to cooperation between academia and government in CITY NAME**?

a. Is there a **continuous exchange** between academia and policymakers in …?

If **yes**, could you give me an overview of what is going on including perhaps some **examples**?

If **no**, in your opinion, **why** is that the case?

b. How **useful** is this exchange in your eyes?
c. Do you think it needs to be **improved**?

   And if **yes**, **why** would you think that and

   in your opinion, **how** should this be done?

5. Perhaps I missed something important in this broad topic of urban governance for health, wellbeing, and equity in your city. **Would you like to add something**?

### Outro

**Thank you very much for all your insights**. The team will now transcribe our conversation and afterwards start to analyze it. Based on the findings from these interviews, we will identify and analyze key challenges and facilitating factors to apply good governance for health and wellbeing.

The results of the study will be fed into a report, including a research protocol and implementation strategy, that will be submitted to the contracting authority. Please contact us or the contracting authority for a summary of the results.

**If you have any questions** that emerge after this conversation, please do not hesitate to contact us.
